# Journal of clinical monitoring and computing 2014 end of year summary: near infrared spectroscopy (NIRS)

**DOI:** 10.1007/s10877-015-9689-4

**Published:** 2015-03-26

**Authors:** Thomas W. L. Scheeren, Karim Bendjelid

**Affiliations:** 1 Department of Anaesthesiology, University Medical Center Groningen, Groningen, The Netherlands; 2 Department of Anaesthesiology and Intensive Care, Intensive Care Service, Geneva University Hospitals, University of Geneva, Geneva, Switzerland; 3 Department of Anaesthesiology, University of Groningen, Groningen, The Netherlands

Near infrared spectroscopy (NIRS) is a non-invasive method for monitoring hemoglobin oxygen saturation within the microvessels. It is therefore also sometimes (erroneously) referred to as a measure of tissue oxygenation. By using self-adhesive optodes on the body surface, measurements can be taken in tissues below the skin up to a depth of 3–4 cm. The near infrared light (unlike visible light) even penetrates bone tissue, allowing measurement of regional cerebral oxygenation (SrcO_2_). There are numerous devices using NIRS technology available on the market, and the main applications for use include monitoring of SrcO_2_ during cardiothoracic and vascular (particularly carotid artery) surgery, as well as non-cardiac surgery that is performed in the sitting or so-called beach chair position [1]. Another widespread use is the assessment of SrcO_2_ in neonates and preterm infants [2]. Furthermore, there have been attempts to use peripheral tissue oxygenation (SptO_2_) measurements by NIRS for guiding therapy and predicting outcome in emergency medicine (both pre-hospital and emergency room use) and in the perioperative setting [3]. Finally, the ability to measure SptO_2_ at different sites [4] raised interest in researchers to look at physiological changes, e.g. during exercise, in musculature of the leg or arm. For this, a special provocation test (the vascular occlusion test (VOT) by inflating a tourniquet proximal to the measurement site) has been developed. The VOT looks at the rate of deoxygenation during ischemia (vascular occlusion), which is considered as a measure of muscle and mitochondrial oxygen consumption, and at the rate of reoxygenation during reperfusion, reflecting the reactivity of the microcirculation [5].

The Journal of Clinical Monitoring and Computing (JCMC) has become an ideal platform for publishing NIRS-related research, as reflected by an increasing number of articles published in the recent years. This article will review two papers on peripheral (SptO_2_) and two papers on cerebral hemoglobin oxygen saturation (SrcO_2_) published last year in the JCMC.

## NIRS for measuring peripheral hemoglobin oxygen saturation

The first two articles focus on a comparison of different devices measuring SptO_2_. Why is that an issue at all? Let’s go back in time: Twenty years ago, with the first appearance of pulse oximeters in clinical use to measure peripheral arterial oxygen saturation (SaO_2_), there were several companies that developed and marketed such devices, which in turn incorporated different technologies (regarding wavelength used, measurement site etc.). As a consequence, at that time SaO_2_ had to be interpreted in view of the device used, and their readings could differ by several percentage points in individual patients. Nowadays, after standardization and technical progress of pulse oximeter technologies, no one cares any longer with what device a given SaO_2_ is measured, since variation between different vendors has become negligible. With NIRS technology, this standardisation process has still to be initiated, and we are currently confronted with a huge variety of devices measuring regional hemoglobin oxygen saturation by using (one to four) different wavelengths, and come with multiple shaped optodes or sensors intended to be used at different anatomic regions (forehead, thenar eminence, somatic organs, muscle etc.). This creates an area of validation research of different NIRS applications, two examples of which are summarized in the following chapter.

The first study compares the use of two NIRS devices on SptO_2_ on the leg muscles of healthy human volunteers, with and without performing exercise [6]. Of note, only one of these devices (the InSpectra, Hutchinson Technologies) is proposed for this application, while the other one (the Invos 4100, Somanetics) is primarily used for measuring SrcO_2_. Consequently, due to these different applications, the authors found a significant difference in muscle hemoglobin oxygen saturation of slightly more than 10 % already at baseline (resting conditions), with the InSpectra showing the higher values (87 ± 8 vs. 76 ± 6 %). The former was in close agreement with baseline values (86 ± 6 %) observed with the same device in 160 patients under general anaesthesia [7]. During exercise, muscle hemoglobin oxygen saturation decreased immediately with both devices, to values close to zero (InSpectra) or slightly above 20 % (Invos) [6]. This decrease was reversible after cessation of exercise, with slight hyperaemia of 8 % (InSpectra) and 18 % above baseline values (Invos), respectively. The authors also performed a vascular occlusion test, which revealed similar results as the exercise test. Finally, they looked at the influence of skin and subcutaneous tissue thickness as assessed by ultrasound on the measurements of both devices and found that the InSpectra, but not the Invos, was prone to increased skin and subcutaneous tissue thickness as reflected by lower SptO_2_ values. Since the measuring depth within a tissue is determined by the distance between the light-emitting and light-detecting part of the optode, the smaller distance within the InSpectra sensor (30 mm as compared to 40 mm in the Invos sensor) might explain its higher susceptibility to thicker skin and subcutaneous tissue. The authors conclude that the Invos, although not primarily designed for this purpose, can be used to detect experimentally and exercise induced skeletal muscle ischemia in the human leg, interchangeable with the InSpectra in this respect [6]. However, the study confirms the abovementioned differences in sensitivity or calibration between NIRS devices to detect changes in regional hemoglobin oxygen saturation.

In the second study looking at peripheral hemoglobin oxygen saturation the authors compared the ability of three NIRS devices (Invos 5100C, Nonin Equanox 7600, and Fore-Sight, CASMed), all of which are primarily marketed for cerebral and somatic use, to measure SptO_2_ on the forearm muscles of healthy human volunteers. [8] The median readings of the three devices at baseline were within 6 % points, with Invos giving the highest and Fore-Sight the lowest steady state values. To test the repeatability of the measurements the sensors were repositioned 20 times on the forearm during hemodynamic steady state conditions. The repeatability of Fore-Sight was best, while that of Equanox was worst, with no difference between same-site and various-site repeatability. Also, the authors repeatedly performed a VOT to induce maximal tissue deoxygenation and to evaluate the dynamic properties of the three NIRS devices. The slopes of desaturation were steepest with Equanox. Furthermore, Equanox and Invos often reached their lower boundary values during ischemia at a level of 20–30 % while Fore-Sight showed continuing desaturation, likely best representing truly changing oxygenation. This difference in dynamics found in SptO_2_ measurements on the arm might also apply to measurements on the forehead, suggesting that differences in so-called removal of extra-cranial tissue contamination as presented in a recent study [9] could be simply a matter of different sensitivity of NIRS devices to overall oxygenation changes in the tissue, as elegantly discussed by the authors. The study has several clinical implications and consequences when it comes to choosing a particular NIRS device: when NIRS oximetry is intended to be used for trend monitoring, the repeatability of measurement is less important. If in addition the sensitivity of a NIRS device to changes in hemoglobin oxygen saturation is low, the risk of undetected true hypoxia will be high. However, if NIRS is to be used as a spot measurement (e.g. in the emergency room) or if the monitoring is started when the patient status is uncertain (e.g. in the ICU or OR in a deteriorating patient without having a baseline value), a good repeatability of measurement or a close proximity to absolute (true) values becomes more important. Since high sensitivity comes at the expense of good repeatability, a pragmatic approach would be to care less about the closeness to the ‘true’ values, but to settle for the device with the best combination of repeatability and sensitivity to changing oxygenation [8].

## NIRS for measuring cerebral hemoglobin oxygen saturation

The most commonly used application for NIRS technology is monitoring cerebral hemoglobin oxygen saturation (SrcO_2_). A recently developed use includes patients undergoing arthroscopic shoulder surgery in the sitting position, which is preferred in some centers despite the danger of hemodynamic instability for the sake of better surgical conditions. Although it is known that SrcO_2_ is lower under those conditions as compared to the alternative lateral decubitus position, the clinical relevant question arises whether intravenous anesthesia or inhalational anesthesia is superior when it comes to preserving cerebral oxygenation. This question was investigated in a recent trial, in which 40 patients were randomized to receive either propofol or desflurane, both titrated to similar anesthetic depth as obtained by bispectral index (BIS), resulting also in similar hemodynamics [10]. While SrcO_2_ significantly decreased in both groups in the first minutes after patients were located from the supine to the sitting position, values remained significantly higher in the desflurane group as compared to the propofol group, despite lower blood pressures. Furthermore, cerebral desaturations, defined as SrcO_2_ below 75 % of individual baseline values, occurred in two patients of the propofol group but in none of the desflurane group. Unfortunately, measurements were stopped 9 min after positioning of the patients, preventing conclusions on the intraoperative course of SrcO_2_ as well as on-going differences between anesthetic agents. The authors conclude that desflurane preserved cerebral oxygenation better than propofol at equipotent anesthetic concentrations [10]. These observations are surprising at first sight, since it is long known that volatile anesthetics impair autoregulation of cerebral blood flow more than propofol [11]. The authors speculate that desflurane (unlike propofol) induced cerebral vasodilation and thus increased cerebral blood flow (CBF), which in turn compensates the reduction in CBF following installation of the sitting position. Otherwise, we may also speculate that the negative impact of propofol on venous return and thus cardiac output is huge in the present posture due to the venodilator effect of the drug.

Another example of the clinical applicability of NIRS technology to measure SrcO_2_ is presented in a case series of 20 patients undergoing major abdominal or urological surgery under general anaesthesia [12]. The authors used controlled hypotension induced by nitroglycerin (NTG) in order to minimize blood loss and optimise surgical conditions. Vasodilation with NTG reduced MAP by about 20 % (from 90 ± 14 to 72 ± 14 mmHg), whereas it increased SrcO_2_ in all patients by an average between 15 and 21 %. Although the authors did not measure blood flow (e.g. cardiac output) and did not report what NIRS device they used, these findings demonstrate that blood pressure and tissue perfusion are not always related in a linear, positive fashion (uncoupling phenomenon). The authors discuss as a potential confounder that the volatile anaesthetics used might have led to luxury perfusion of the monitored brain area while diverting blood away from potentially ischemic regions. [12] Nevertheless, this case series shows that NIRS monitoring can be used to ensure the adequacy of tissue oxygenation during controlled hypotension and expands the scope of conventional hemodynamic monitoring in this respect. This way it may contribute to improve patient’s safety in case of macro-microcirculatory decoupling.

## Interpretation of SrcO_2_ values

Since there is no consensus about what should be considered “normal values” for cerebral hemoglobin oxygen saturation, the authors of the previous study [8] come up with a very interesting calculation: Assuming that a jugular bulb saturation (SjbO_2_, the gold standard for assessing cerebral hypoxemia) below 50 % indicates cerebral ischemia, then with a ratio between arterial and venous blood volumes of 25:75 contributing to the SrcO_2_ signal displayed on the monitor, as most NIRS devices adopt, we can calculate which readings actually indicate cerebral ischemia and which readings may assure us that this is not the case [8]. In detail, if the SaO_2_ is 95 % and SjbO_2_ 50 %, the resulting SrcO_2_ should be 61 %, which can be considered as “normal”. Thus with a standard deviation of 5 % a SrcO_2_ measurement should be below 52 % (61−1.96 × 5 %) to be more than 95 % certain that the tissue is actually ischemic and above 71 % (61 + 1.96 × 5 %) to be certain that it is not, implying a large “grey zone” of SrcO_2_ values where its diagnostic ability is inconclusive [13]. On the other hand, changing of the body position (e.g. head-up or head-down tilting) may change the ratio between arterial and venous blood volumes of 25:75, so that for instance decreases in the SrcO_2_ readings are simply the results of an increased proportion of venous blood contributing to the signal, rather than a true change in cerebral tissue oxygenation. In this respect, the higher SrcO_2_ values after raising patients to the sitting position during desflurane anesthesia in the abovementioned study [10] could be interpreted as evidence for cerebral arterial vasodilation under desflurane and maintained cerebrovascular tone under propofol anesthesia.

In conclusion, until a standardization of NIRS devices has been effectuated, the clinical context may influence the choice of the adequate NIRS device for a specific purpose.
